# Identifying disease-related microbes based on multi-scale variational graph autoencoder embedding Wasserstein distance

**DOI:** 10.1186/s12915-023-01796-8

**Published:** 2023-12-20

**Authors:** Huan Zhu, Hongxia Hao, Liang Yu

**Affiliations:** School of Computer Science and Technology, Xidian University, Xi’an, China

**Keywords:** Variational graph autoencoder, Wasserstein distance, Microbe-disease association, XGBoost

## Abstract

**Background:**

Enormous clinical and biomedical researches have demonstrated that microbes are crucial to human health. Identifying associations between microbes and diseases can not only reveal potential disease mechanisms, but also facilitate early diagnosis and promote precision medicine. Due to the data perturbation and unsatisfactory latent representation, there is a significant room for improvement.

**Results:**

In this work, we proposed a novel framework, Multi-scale Variational Graph AutoEncoder embedding Wasserstein distance (MVGAEW) to predict disease-related microbes, which had the ability to resist data perturbation and effectively generate latent representations for both microbes and diseases from the perspective of distribution. First, we calculated multiple similarities and integrated them through similarity network confusion. Subsequently, we obtained node latent representations by improved variational graph autoencoder. Ultimately, XGBoost classifier was employed to predict potential disease-related microbes. We also introduced multi-order node embedding reconstruction to enhance the representation capacity. We also performed ablation studies to evaluate the contribution of each section of our model. Moreover, we conducted experiments on common drugs and case studies, including Alzheimer’s disease, Crohn’s disease, and colorectal neoplasms, to validate the effectiveness of our framework.

**Conclusions:**

Significantly, our model exceeded other currently state-of-the-art methods, exhibiting a great improvement on the HMDAD database.

## Background

Microorganisms are a class of microscopic organisms that exist in the form of single cells or colonies [1]. Extensive research has confirmed the close interaction between human hosts and the majority of microbial colonies, which mostly consist of bacteria, archaea, viruses, and protozoa [2, 3]. Microorganisms are commonly present on and within various human body organs, such as the mouth, skin, and intestines. Particularly, the majority of these microorganisms are located within the gastrointestinal tract [4]. Actually, the majority of commensal microorganisms inhabiting humans are not detrimental to health and even have mutually beneficial relationships with their human hosts [5]. The human microbiome is usually perceived as the “humanity’s forgotten organ” due to its liver-like abilities, including promoting nutrient absorption, resisting the invasion of pathogens, and promoting metabolism [6–8]. There has reached a consensus that dysbiosis or imbalance in microbial communities can lead to human disease [9, 10], such as asthma [11], diabetes [12], and cancer [13]. For instance, the overgrowth of Klebsiella bacteria in the gut has been shown to play a role in several chronic diseases, including colitis and Crohn’s disease [14]. Conversely, following a low-starch diet can help impede the growth of Klebsiella bacteria and thus, potentially alleviate symptoms of Crohn’s disease [15]. Therefore, identifying associations between microbes and diseases can not only reveal potential disease mechanisms, but also facilitate early diagnosis and promote precision medicine through potential biomarkers. Considering that traditional biomedical experiments are time and labor consuming, it is critical to develop computational methods with high accuracy and efficiency for microbe-disease association prediction.

In recent years, a multitude of computational methods has been proposed to predict microbe-disease associations. These methods can be roughly categorized into four groups: network-based methods, matrix factorization methods, regularization methods, and neural network methods, as mentioned by Wang et al. [16] and Wen et al. [17]. (1) The first category was the most intuitionistic method with strong interpretability, which adopted topological information from networks constructed using multiple databases. For example, Chen et al. [18] proposed KATZHMDA based on the KATZ measure for predicting microbe-disease association, while Lei et al. [19] designed LGRSH, which implemented node2vec algorithm [20] to obtain the low-dimensional representations and adopted the improved rule-based inference method for microbe-disease association prediction. (2) The core idea of matrix factorization methods is factorizing the input matrix into two matrixes of lower dimensionality, which simultaneously maintain the property of reconstruction. RNMFMDA, proposed by Peng et al. [21], employed random walk with restart to achieve reliable negative sampling on the microbe-disease network and subsequently employed a neighborhood regularized logistic matrix factorization technique to predict the likelihood of microbe-disease associations. (3) Regularization methods are characterized by their application to least square classifications using different forms of regularization. Typically, Xu et al. [22] proposed MDAKRLS by combining hamming interaction spectral similarity with Kronecker regularized least squares for microbe-disease association prediction. (4) Neural network methods prevailed over other methods by miles. Long et al. [23] designed a new framework named GATMDA, to represent microbes and diseases and predict associations based on an optimized graph attention network with inductive matrix completion. Furthermore, MVGCNMDA, proposed by Hua et al. [24], utilized the multi-view graph for data augmentation and multi-channel attention to predict disease-related microbes.

Despite the promising progress made by the aforementioned methods, there are still some limitations and shortcomings. Firstly, the most vital point is the perturbation, including noise and deficiency, in similarity networks or other heterogeneous networks, which is usually caused by the incomplete data or the bias of network construction means. Secondly, merely considering a similarity network from a single perspective may result in information insufficiency. Meanwhile, the simple averaging of similarity networks from different perspectives seems too naïve and how to reasonably aggregate similarity networks is still challenging. Thirdly, we observed that models with strong interpretation generally performed unsatisfactorily, whereas some models with lower interpretation, especially in neural network methods, performed better, indicating the capacity of latent representation needs to be improved.

Taking the above limitations into consideration, in this work, we proposed a novel framework, Multi-scale Variational Graph AutoEncoder embedding Wasserstein distance (MVGAEW) for identifying disease-related microbes. Firstly, we calculated disease and microbe similarities from different perspectives, including disease functional similarity, microbe functional similarity, and Gaussian interaction profile kernel similarity. Further, we integrated different similarity matrixes by leveraging similarity network confusion (SNF [25]). Secondly, we introduced the variational graph autoencoder (VGAE [26]) to learn node latent representations. The Wasserstein distance(WD [27]) and the idea of multi-scale [28] were employed to improve the representational capacity of VGAE. Moreover, inspired by the diffusion model [29] and parallel neighborhood reconstruction [27], we innovatively proposed an auxiliary task, multi-order node embedding reconstruction, to enhance the robustness of VGAE. Ultimately, we utilized XGBoost [30] to predict the potential microbe-disease pairs by inputting the concatenation of latent representations for each microbe and disease. Our experimental results on the HMDAD database indicated that our proposed model exceeded other current SOTA methods with a great promotion. Significantly, we also conducted validations based on common drugs and several case studies on Alzheimer’s disease, Crohn’s disease, and colorectal neoplasms, which further validate the effectiveness of MVGAEW.

## Results and discussion

### Experiment settings

In this study, tenfold cross-validations were adopted to ensure the accuracy and reliability of our model. We conducted a series of frequently used metrics from multiple perspectives, including AUROC, AUPR, F1, Precision, Recall, and Accuracy, to evaluate our model’s performance across all comparison experiments. In the SNF part, we set the number of neighbors in KNN as 5 and 30 for diseases and microbes in the HMDAD database. In the VAGE part, we used three scales of multi-scale encoders for both disease and microbe similarity networks, including 16, 32, and 64. In addition, the parameters of the XGBoost classifier were set as default. We adopted the StepLR strategy to schedule the learning rate during training, in which the learning rate will be progressively updated until it reached the specified epochs.

### Ablation study

To provide a detailed analysis of the contribution of each component in VGAE, we carried out ablation experiments based on the HMDAD database. MVGAEW refers to the complete model without any components removed. Del_WD denotes the model without the WD component, replaced with KL-divergency. Del_multi-scale represents the model without a multi-scale layer in the encoder portion. Del_aux_1 and Del_aux_2 represent the model without the auxiliary 1st-order and 2nd-order node embedding reconstruction tasks, respectively. Literally, Del_aux_1_2 indicates the model taking no account of auxiliary task. Through these experiments, we aimed to analyze the individual contribution of each component towards the overall model accuracy and performance Table 1.

**Table 1:** Performance of ablation experiments based on the HMDAD database

Method	AUROC	AUPR	F1	Precision	Recall	Accuracy
**MVGAEW**	**0.9798**	**0.9855**	**0.9412**	**0.9524**	0.9302	**0.9444**
**Del_WD**	0.9446	0.9419	0.8842	0.8077	**0.9767**	0.8778
**Del_multi-scale**	0.9684	0.9715	0.9111	0.8723	0.9535	0.9111
**Del_aux_1**	0.9746	0.9789	0.9091	0.8889	0.9302	0.9111
**Del_aux_2**	0.9749	0.9808	0.9213	0.8367	0.9534	0.9222
**Del_aux_1_2**	0.9737	0.9799	0.8913	0.8367	0.9530	0.8889

The bold values denote the max value in columns

As shown in Table 2, we notice that almost each experiment with the prefix Del does not perform as well as MVGAEW, indicating the three major ideas integrated into our model are effective. In terms of AUROC and AUPR, the sharply reduced experiment is Del_WD, verifying the contribution brought from WD is more than KL-divergency and other major ideas, which is also consistent with the point that bottleneck lies in the disappearance of gradient information from KL-divergence during later stages of training. Similarly, the second sharply reduced experiment is Del_multi-scale with a decreasing percentage of 1.164%, revealing that the strategy of the multi-scale encoder is effective. Compared to Del_aux_1_2, Del_aux_1 and Del_aux_2 both demonstrate improved performance except Recall, suggesting that either 1st-order or 2nd-order node embedding reconstruction tasks can be valid. Furthermore, the degree of decline of Del_aux_1 is greater than that of Del_auu_2, highlighting the importance of 1st-order node-wise feature information over the 2nd-order counterparts.

**Table 2:** The comparison between our model and other methods under tenfold cross-validations on the HMDAD database

Method	AUROC	AUPR	F1	Precision	Recall	Accuracy
**MVGAEW**	**0.9798**	**0.9855**	**0.9412**	0.9524	0.9302	0.9444
**GATMDA**	0.9398	0.9364	0.8151	0.8672	0.7689	0.8256
**RNMFMDA**	0.9124	0.2767	0.1297	0.0753	0.4667	**0.9732**
**KATZHMDA**	0.8348	0.5910	0.2017	0.1160	0.7733	0.7482
**LRLSHMDA**	0.8851	0.6080	0.2243	0.1290	0.8600	0.7553
**MVGCNMDA**	0.9196	0.9237	0.9113	**0.9843**	0.8484	0.9178
**MVFA**	0.9718	0.8864	0.8755	0.7961	**0.9729**	0.8622

The bold values denote the max value in columns

### Performance comparison with SOTA methods

To evaluate the effectiveness of our proposed model, we conducted several comparative experiments against classical representative prediction approaches. Within these experiments, we compared some representative methods from Matrix Factorization, Regularization, and Neural Network, As previously mentioned by wang et al. [16] and Wen et al. [17]. The brief summarization is shown as follows:

KATZHMDA [18], the first proposed method for the prediction of microbe-disease associations, utilized KATZ measurement to calculate the node centrality for prediction.

RNMFMDA [21], which integrated reliable negative sampling into neighborhood regularized logistic matrix factorization to evaluate the likelihood of associations for all microbe-disease pairs.

LRLSHMDA [31], which featured with the least squares classifier with Laplacian regularization to solve the link prediction task.

GATMDA [23], incorporated the concept of “talking heads” into the optimized graph attention network to learn latent representations from microbes and disease.

MVGCNMDA [24], which analogously adopted the idea of multi-scale and utilized the multi-view graph for data augmentation to predict disease-related microbes.

MVFA [32], which proposed a multi-view feature aggregation model that combines both linear and nonlinear features to recognize disease-related microbes.

The comparison experiments were scheduled under tenfold cross validations based on HMDAD database. In addition, we also carried out parameter adjustment experiments for each of the implemented methods to ensure that their performance was as close as possible to that reported in their original papers.

**Fig. 1:**
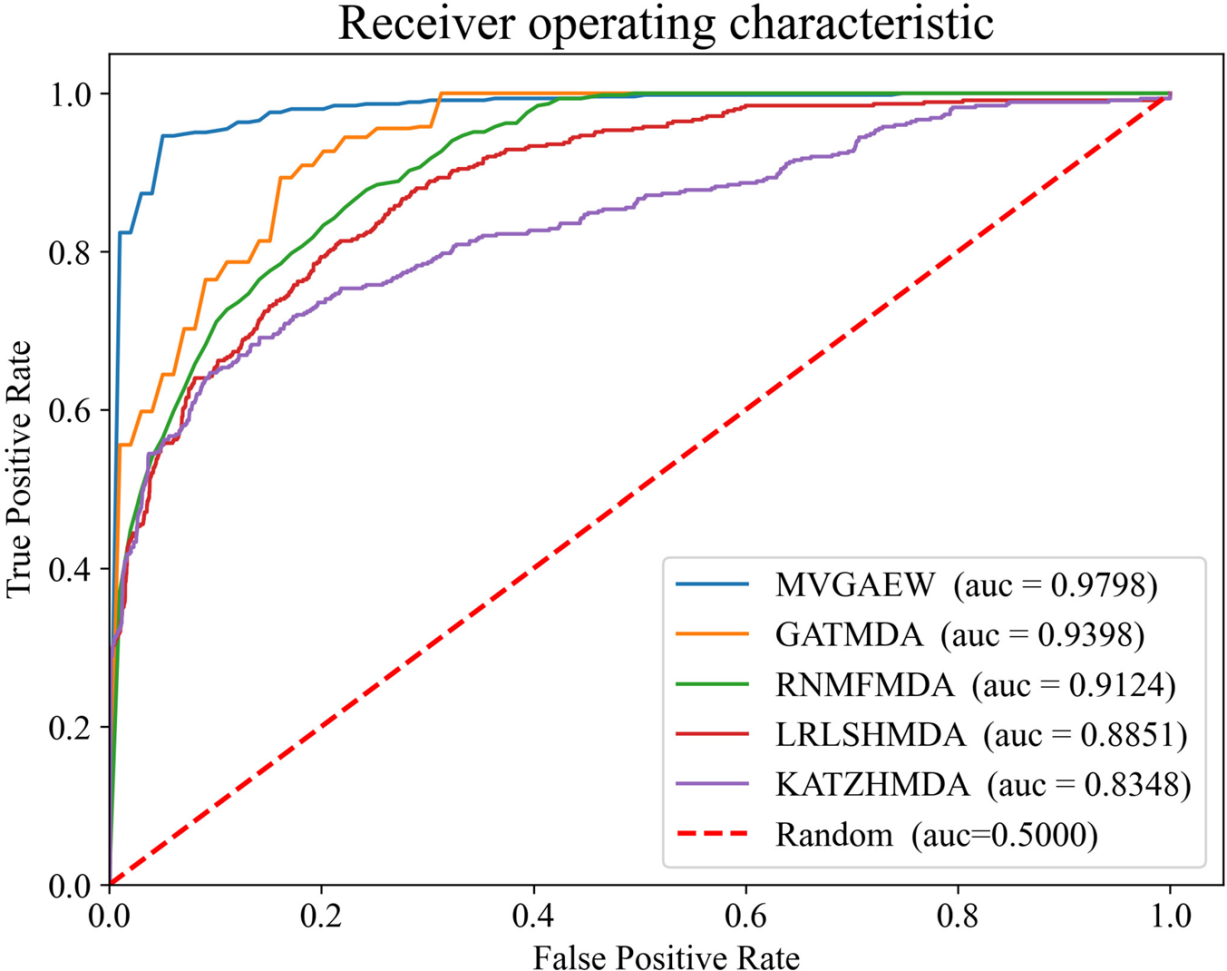
The ROC curves of different models on tenfold cross-validations

**Fig. 2:**
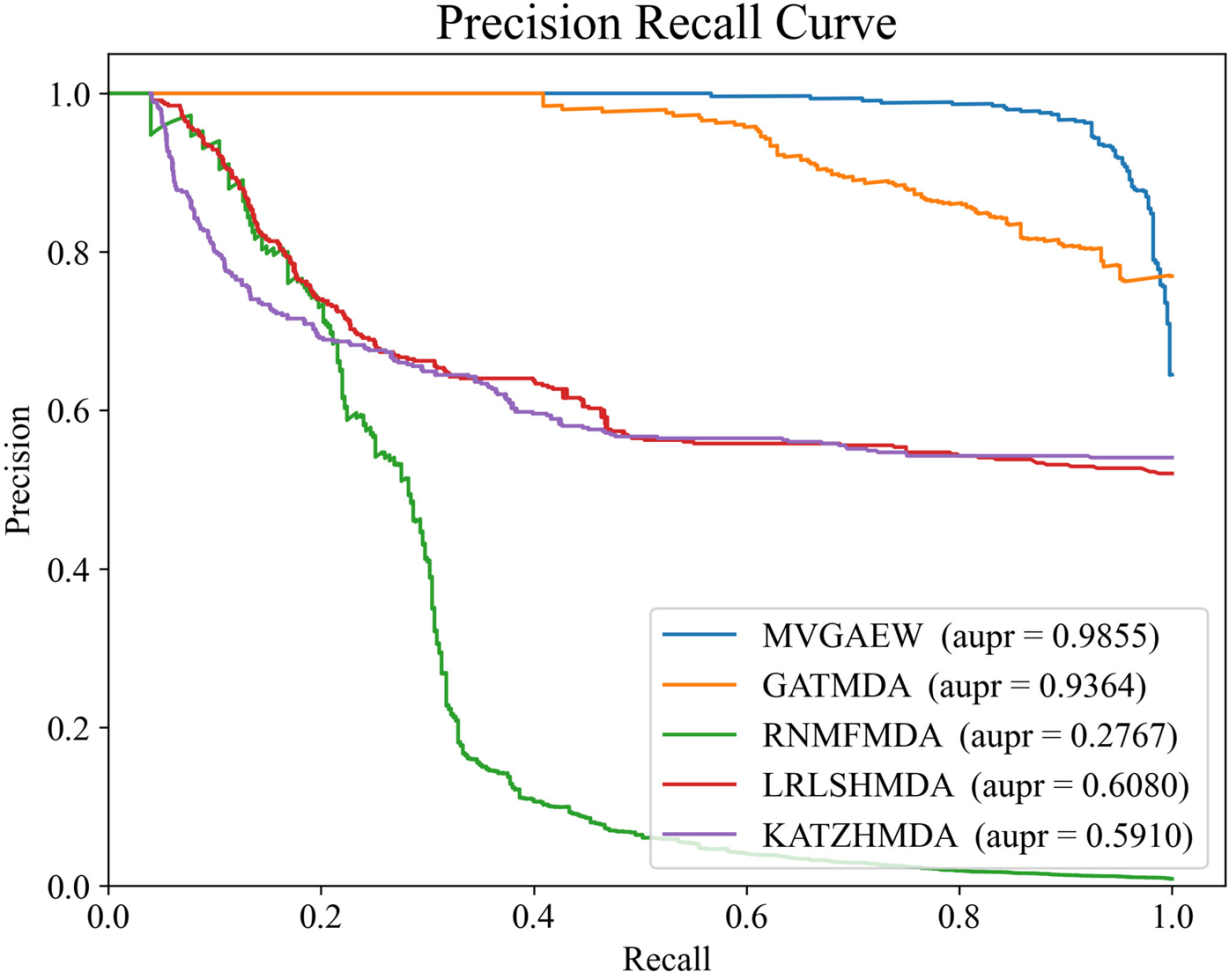
The PR curves of different models on tenfold cross-validations

As shown in Figs. 1 and 2, our proposed model achieves higher AUROC and AUPR scores compared to other methods, demonstrating its superior performance. Furthermore, the performance of different methods across multiple metrics is demonstrated in Table 2. It is obvious that the F1 value of our model also dominates other approaches. Despite the precision and recall values of our model not being the highest, the balance between precision and recall is fabulous in a higher level, rather than the large gap in a lower level like that in LRLSHMDA, KATZHMDA and RNMFMDA. As well-known, the F1 metric is designed to make a tradeoff between precision and recall and is considered a splendid metric to measure the performance of the model, which is consistent with the fact that the F1 value of our model exceeds others. It is also evident that the traditional methods, such as LRLSHMDA, KATZHMDA, and RNMFMDA, perform poorly, while other neural network methods show superior performance. In addition, we note that the accuracy of our model ranks second, with RNMFMDA achieving the best performance. It is worth noting that RNMFMDA adopted a reliable negative sampling strategy, resulting in the negative samples fed into the model being quite simple and leading to the trained model tended to learn simple knowledge and local distribution. Furthermore, this also can be verified in the lower AUPR and precision scores, which are metrics that focus on negative samples.

### Performance comparison with widely used databases

As the accumulation of data, databases become more mature, containing increasingly valid associations between microbes and diseases. To ensure scalability and powerful generalization, we conducted several experiments based on three additional databases. Giving enough thought to the sparse matches of microbes between the microbe-disease database and the microbe-drug database, we calculated the microbe similarities for the latter database without relying on drug-based functional similarity.

As shown in Table 3, our model based on three additional databases also performs well. Apart from HMDAD, the most impressive results come from Peryton, the latest published database, with the highest density of known association networks. We observed that model performance improves over time as the databases increase in both quality and quantity, and their distribution becomes more representative of the true global distribution.

**Table 3:** The comparison of all microbe-disease databases under tenfold cross validations

Database	AUROC	AUPR	F1	Precision	Recall	Accuracy
**HMDAD**	**0.9798**	**0.9855**	**0.9412**	**0.9524**	0.9302	**0.9444**
**Disbiome**	0.9451	0.9388	0.8761	0.8590	0.8939	0.8717
**MicroPhenDB**	0.9616	0.9576	0.8899	0.8779	0.9022	0.8902
**Peryton**	0.9668	0.9630	0.9013	0.8726	**0.9320**	0.9029

The bold values denote the max value in columns

### Interpretation of latent representation

Our model has undeniably demonstrated outstanding performance for the microbe-disease associations prediction task. With the purpose of further exploring the interpretability of latent representation from the insight of distribution, we visualized the feature distribution of the adopted latent representation for microbes. Specifically, we accomplished this by employing the t-SNE [33] method to project high-dimensional data into a two-dimensional (2D) plane for visualization.

**Fig. 3:**
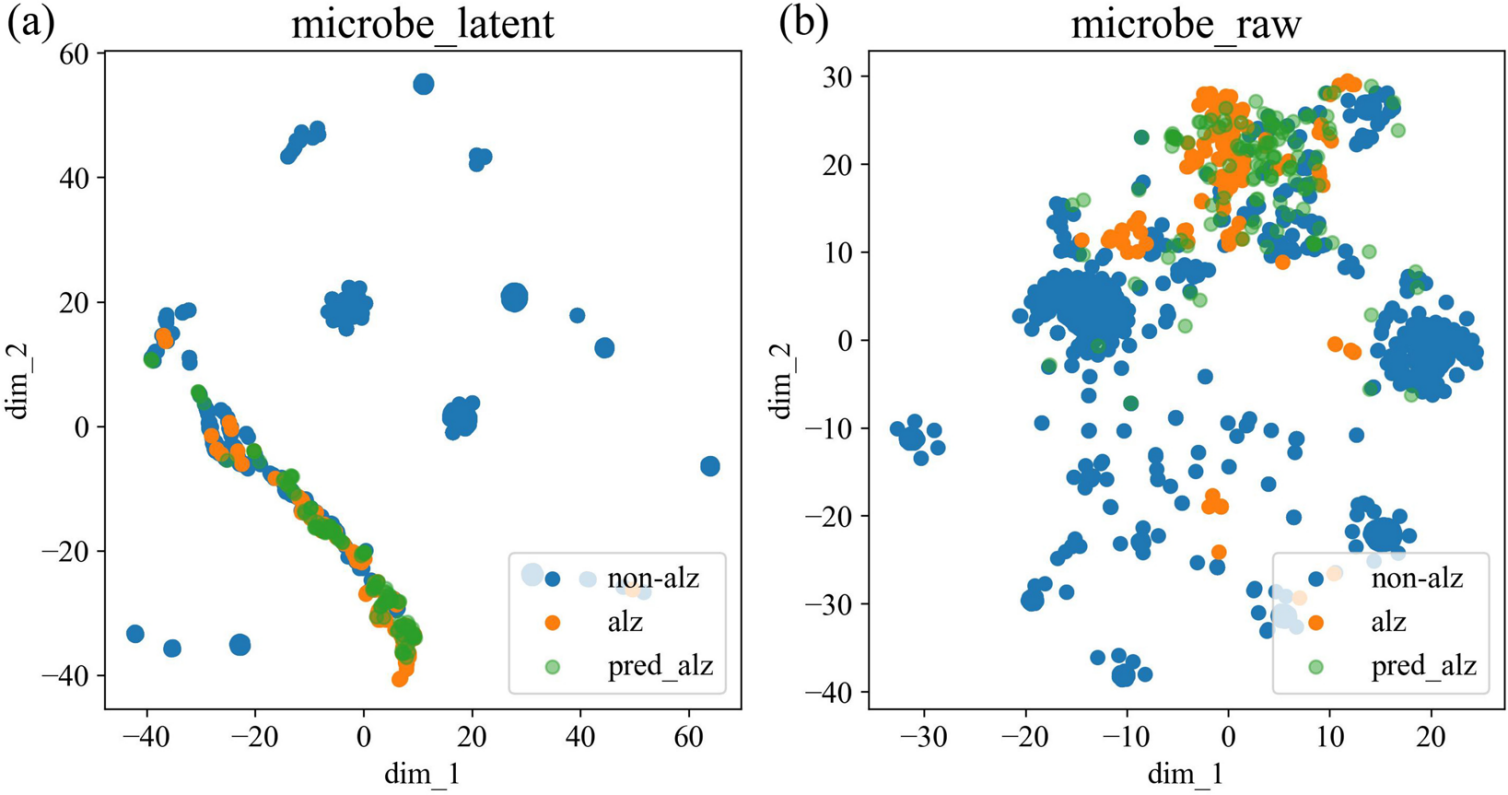
Visualizations of distribution whether adopting latent representation for microbes related to Alzheimer’s disease. **a** The latent distribution by adopting latent representation proposed in our framework and **b** the raw distribution of integrated similarity network

Figure 3a demonstrates the distribution after adopting latent representation, while Fig. 3b shows the distribution of raw integrated similarity network without the use of latent representation. The points labeled as “alz” and “non-alz” on both figures indicate whether a particular microbe is related to Alzheimer’s disease [34] in the peryton database, while the points labeled as “pred_alz” in both figures represent the potential microbes that MVGAEW predicts to be related to Alzheimer’s disease within the top 50 probabilities. The clusters in Fig. 3a are clearly more tightly packed and exhibit a pattern characterized by long strips associated with specific diseases. However, some points labeled as “pred_alz” in Fig. 3b are completely disconnected from known associations, suggesting that microbes with a high probability may not be identified if the integrated similarity network is used alone, without employing other representation learning methods.

### Validation based on common drugs

Subsequently, with the purpose of further exploring the validity of our model, we investigated common drugs related to specific microbes and diseases. It is well-known that specific drugs can impact diseases and interfere with microbial metabolism. Spontaneously, there may be a strong association between a disease and a microbe if they do share common related drugs. To further support the potential association between a disease and a microbe, we conducted literature verification in Pubmed to identify any relevant explanations or studies regarding the specific microbe-disease pair.

We obtained disease-related drugs by utilizing the MalaCard database [35], which is an integrated and continuously updated database of human diseases and their annotations from 75 data sources. To extract microbe-related drugs, we utilized both the MDAD and aBiofilm databases, which contain high-confidence microbe-drug associations. To maximize the number of microbe-related drugs obtained, we mapped microbes of MicroPhenDB with those in MDAD and aBiofilm. We presented the probabilities predicted by MVGAEW between a given microbe-disease pair in Table 4, along with corresponding PubMed IDs (PMID). As expected, the pairs with higher probabilities shared more common drugs, which is in line with the observation that disease-related drugs tend to impact multiple microbes. For instance, in the case of colorectal cancer, tobramycin has been shown to impact both *Escherichia coli* and *Staphylococcus aureus*.

**Table 4:** The common drugs related to specific microbe and disease

Microbe	Disease	Common drugs	Probability	PMID
*Escherichia coli*	Non-alcoholic fatty liver disease	Sorbitol, rifampicin	0.9491	31,808,577
*Escherichia coli*	Colorectal cancer	Ertapenem, tobramycin, framycetin	0.9474	28,106,826
*Escherichia coli*	Atopic eczema	Zinc oxide, tannic acid	0.9403	33,023,370
*Escherichia coli*	Cirrhosis of liver	Imipenem, cefoperazone, cefoxitin	0.8973	31,295,531
*Escherichia coli*	Hiv infection	Sulfamethoxazole	0.8920	25,482,819
*Escherichia coli*	Mouth neoplasm	Sorbitol	0.8245	35,096,312
*Staphylococcus aureus*	Colorectal cancer	Azithromycin, tobramycin	0.7997	24,467,507
*Escherichia coli*	Bacterial vaginosis	Tetracycline, tannic acid	0.7771	29,933,767
*Escherichia coli*	Congenital short bowel syndrome	Daidzein	0.7081	9,125,641
*Staphylococcus aureus*	Cirrhosis of liver	Imipenem, azithromycin, cefoxitin	0.6987	22,833,245
*Staphylococcus aureus*	Non-alcoholic fatty liver disease	Rifampicin	0.6948	34,978,141
*Escherichia coli*	Dental caries	Sorbitol	0.6351	30,657,107
*Escherichia coli*	Sclerosing cholangitis	Curcumin, minocycline	0.6282	30,252,934
*Escherichia coli*	Otitis media	Cefpodoxime	0.6270	28,613,732
*Staphylococcus aureus*	Periodontitis	Norgestimate, azithromycin, minocycline	0.5752	30,241,716

### Case studies

In this section, we conducted case studies on specific diseases to demonstrate the capability of predicting disease-related microbes. The diseases we focused on include Alzheimer’s disease [34], Crohn’s disease [36], and colorectal neoplasms [37]. Based on the peryton database, we screened out known microbe-disease associations and predicted microbes with probability in the top 20 for each concerned disease. In addition, we also provided corresponding evidence from Pubmed to confirm the existence of these associations.

Alzheimer’s disease (AD) is a prevalent, chronic, and progressive neurodegenerative disease that is considered a kind of dementia. Often characterized by symptoms of memory loss and emotional regulation disorders, weakened learning ability, and loss of motor ability, it can significantly impact the development of individuals, families and even society [38]. As previous works reported, there is a direct link between altered gut microbiota and the development of AD. Furthermore, studies have indicated that AD can be prevented through intermittent fasting [39]. As demonstrated in Table 5, 17 kinds of microbes have the support of literature, while the remainder suggest a strong potential association related to AD. In particular, we further conducted validations on Fusobacteriaceae from multiple perspectives. Through high throughput DNA sequencing, researchers have shown that levels of Fusobacteriaceae are consistently higher, while levels of Prevotellaceae are generally lower, in subjects without dementia [40]. In the aspect of inflammation, Fusobacteriaceae have been found to be strongly associated with inflammation in hepatic encephalopathy [41]. Additionally, high levels of Fusobacteriaceae in the IR-MO group have been found to be associated with low-grade inflammation in adipose tissue among people with insulin resistance and morbid obesity [42]. Simultaneously, Yang et al. [43] suggested that inflammation may be a contributing factor in the progression of AD. Collectively, these findings strengthen the evidence linking Fusobacteriaceae to the development of AD.

**Table 5:** Top 20 predicted microbes related to Alzheimer’s disease

Rank	Microbe	PMID
1	*Fusobacteria*	25,576,662
2	*Roseburia*	35,173,707
3	*Fusobacteriaceae*	Unconfirmed
4	*Megasphaera*	Unconfirmed
5	*Actinomycetaceae*	35,275,538
6	*Fusobacterium*	25,576,662
7	*Klebsiella*	36,068,280
8	*Veillonellaceae*	32,533,776
9	*Butyricicoccus*	36,185,477
10	*Veillonella*	34,931,394
11	*Coprococcus*	35,807,841
12	*Fusobacterium nucleatum*	25,576,662
13	*Corynebacterium*	32,290,475
14	*Campylobacter*	32,290,475
15	*Oribacterium*	Unconfirmed
16	*Faecalibacterium prausnitzii*	34,622,235
17	*Oscillospira*	36,185,477
18	*Citrobacter*	22,891,247
19	*Escherichia coli*	29,472,250

Crohn’s disease (CD), a subtype of inflammatory bowel disease (IBD), is characterized by gut microbiome dysbiosis and accompanied by extraintestinal symptoms such as fever and nutritional disturbance. Colorectal neoplasms (CN), a common malignant tumor in the gastrointestinal tract, are often caused by unhealthy living habits or environmental pollution. Similarly, CN are also characterized by dysbiosis in the gut microbiota [37]. As shown in Tables 6 and 7, we have provided the top 20 predicted microbes and corresponding evidence for both CD and CN for future research. It is important to note that the unconfirmed microbes are supposed to attract more attention in the future studies.

**Table 6:** Top 20 predicted microbes related to Crohn’s disease

Rank	Microbe	PMID
1	*Atopobium*	35,122,247
2	*Barnesiella*	35,806,099
3	*Parasutterella*	35,971,134
4	*Methylobacterium*	33,430,702
5	*Xanthomonadales*	Unconfirmed
6	*Corynebacteriaceae*	25,689,526
7	*Lachnoclostridium*	36,034,848
8	*Leptotrichia*	Unconfirmed
9	*Parvimonas*	34,935,421
10	*Rhodococcus*	25,546,345
11	*Epsilonproteobacteria*	32,040,665
12	*Sphingobacteriia*	Unconfirmed
13	*Enterobacter*	31,764,438
14	*Schwartzia*	3,318,407
15	*Salmonella*	22,009,735
16	*Bradyrhizobiaceae*	Unconfirmed
17	*Ochrobactrum*	Unconfirmed
18	*Halomonas*	Unconfirmed
19	*Halomonadaceae*	Unconfirmed
20	*Bacillaceae*	35,967,326

**Table 7:** Top 20 predicted microbes related to colorectal neoplasms

Rank	Microbe	PMID
1	*Actinomycetales*	33,934,716
2	*Erysipelotrichia*	Unconfirmed
3	*Escherichia coli*	28,106,826
4	*Rothia mucilaginosa*	Unconfirmed
5	*Limosilactobacillus fermentum*	31,581,581
6	*Flavonifractor*	34,799,562
7	*Barnesiella*	32,502,642
8	*Holdemanella*	31,988,379
9	*Erysipelotrichales*	Unconfirmed
10	*Selenomonadales*	Unconfirmed
11	*Erysipelatoclostridium*	35,269,806
12	*Veillonella dispar*	26,549,775
13	*[Clostridium] leptum*	18,237,311
14	*Candidatus Saccharibacteria*	Unconfirmed
15	*Barnesiellaceae*	Unconfirmed
16	*Verrucomicrobia*	34,389,559
17	*Bifidobacterium longum*	31,340,751
18	*Butyrivibrio*	16,317,136
19	*Roseburia faecis*	21,850,056
20	*Comamonadaceae*	28,431,244

**Fig. 4:**
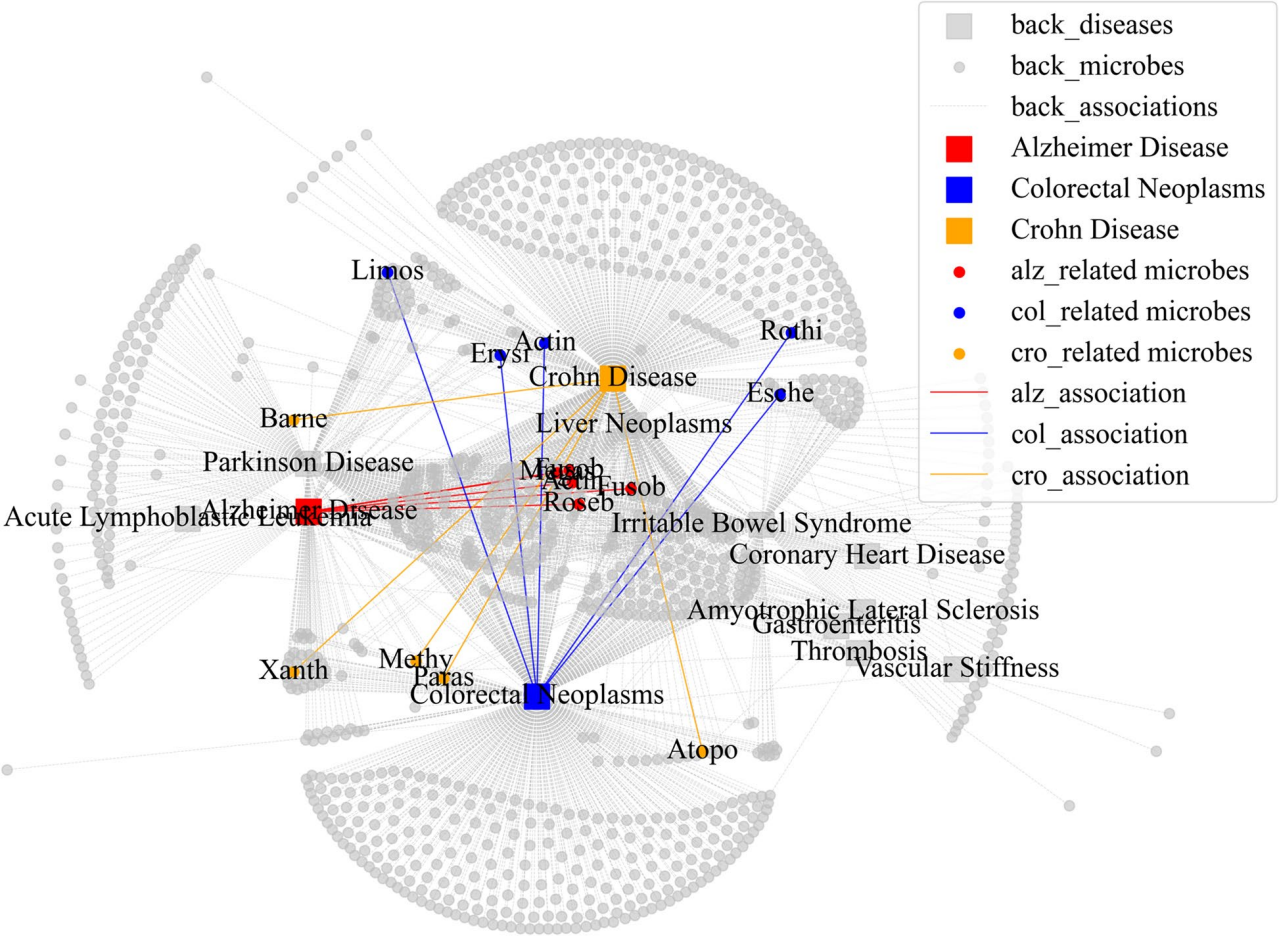
The distribution of existing and predicted associations related to case diseases

Further, we visualized the distribution of existing and predicted associations related to specific diseases as shown in Fig. 4. The four most relevant diseases were screened out for each case disease through an integrated disease similarity matrix and identified the top 5 predicted microbes related to each case disease. In Fig. 4, we observed that the microbes in the center appear to affect multiple diseases, and the predicted microbes further support this finding. For instance, Xanthomonadales was found to be associated with both Parkinson’s disease and CN. We also noticed that there are common microbes shared between CD and CN, as well as a considerable overlap between CD and Parkinson’s disease. Therefore, it is highly likely that Xanthomonadales is related to CD, and this observation further highlights the pattern of second-order neighbors.

## Conclusions

In this study, we proposed a novel framework, named MVGAEW, to identify disease-related microbes. Starting with the point of data perturbation, we utilized VGAE to fit distribution, which allow us to deal with the interference caused by perturbation. VGAE was advantageous in capturing neighbor structure information while mitigating the impact of noise and deficiency to some extent by modeling the true probability distribution. To further enhance the representational capacity of VGAE, we incorporated the multiscale concept to capture local and global patterns at different scales. This allowed us to learn a more complicated probability distribution with high robustness. Additionally, we innovatively designed an effective auxiliary task, called multi-order node embedding reconstruction, to maintain the neighbor embeddings during message propagation. Furthermore, the Wasserstein distance was employed to substitute KL divergence to maintain the gradient information during backpropagation. After calculating and integrating similarity networks, we utilized the improved VGAE for latent representation. Ultimately, XGBoost was adopted to predict the probability between a given pair of microbe and disease. To validate the performance of our model, we carried out several comparison experiments with SOTA methods and performed an ablation study. Most importantly, our approach not only provided the interpretation of latent representation, but also included sufficient validations to verify the effectiveness of our model.

Although outstanding performance has been achieved in several studies, there is still room for improvement. Particularly in handling imbalanced samples, there is a lack of research on generating productive positive samples, which is still a challenging task. It seems meaningless to sample out reliable negative samples, which would perhaps learn a simple distribution and result in overfitting. Relatively, how to generate productive positive samples remains a significant challenge. Furthermore, it is fascinating to predict signed microbe-disease association as the undirected network would lead to loss of information. Last but not least, a promising research direction is the introduction of multi-task learning into the prediction of disease-microbe-drug associations, which can leverage shared structures and potentially enhance the model’s overall performance.

## Methods

### Data sources

#### Microbe-disease association databases

Until now, researchers have developed several widely used databases for microbe-disease association prediction as summarized in Table 8. In 2016, Ma et al. developed the first Human Microbe–Disease Association Database (HMDAD [44]), which collected 450 confirmed microbe-disease associations between 39 diseases and 292 microbes from published literature after redundancy elimination. In 2018, Janssens et al. established Disbiome [45], a database that catalogs 8731 known associations between 1622 microbes and 374 diseases, by screening out from 1191 published academic papers without redundancy. Subsequently, MicroPhenDB [46] was constructed by the same means of HMDAD and Disbiome, including 5511 non-redundant associations between 500 diseases and 1,774 microbes in 22 newly collected human parts. Recently, Skoufos et al. proposed Peryton [47], which was constructed by collecting experimentally supported associations and contained 4172 available associations between 1396 microbes and 43 diseases. We converted the information on known microbe-disease associations into a binary matrix ${\text{A}} \in {{\mathbb{R}}^{nm \times nd}}$ for ease of use, in which the value is 1 if microbe-disease item exists in database, and 0 otherwise. $nm$ and $nd$ represent the number of unique diseases and unique microbes, respectively.

**Table 8:** Databases for microbe-disease association prediction

Database	Microbes	Diseases	Associations	Year
**HMDAD**	292	39	450	2016
**Disbiome**	1622	374	8731	2018
**MicroPhenDB**	1774	500	5511	2020
**Peryton**	1396	43	4172	2021

#### Disease similarity network

In our proposed framework, we adopted three kinds of disease similarity calculation methods: semantic, symptom, and Gaussian interaction profile kernel.

1. Disease semantic similarity (DSS1)

We obtained the disease semantic information from the Medical Subject Headings (MeSH) database. Generally, the semantic information of a disease can be represented by a directed acyclic graph, (DAG) with MeSH descriptors. The formula for the DAG of a disease is typically formulated as $DAG(d) = (d,T(d),E(d))$, where $T(d)$ denotes all related nodes in the DAG of the disease $d$, and $E(d)$ represents all edges in specific DAG.

With the introduction of DAG, Wang et al. [48] exploited the first disease semantic similarity computing method, in which the contribution of each disease $d$ to disease $D$ could be formulated as below:

1 $${C_D}(d) = \left\{ \begin{gathered} \max \{ \Omega \times {C_D}(d^{\prime})|d^{\prime} \in children \, of \, d\} {, }if \, d^{\prime} \ne D, \hfill \\ 1, \, else{.} \hfill \\ \end{gathered} \right.$$

where $\Omega$ represents the contribution factor. Whereafter, the semantic value of a disease $D$ can be aggregated by the semantic contribution of nodes in corresponding DAG, described below:

2 $$V(D) = \sum\limits_{d \in T(D)} {{C_D}(d)}$$

Considering the symmetry, we calculated the semantic contribution for each disease and normalized it by the sum of the semantic values of each disease, described as below:

3 $$DDS1(D1,D2) = \frac{{\sum\nolimits_{d \in T(D1) \cap T(D2)} {({C_{D1}}(d) + {D_{D2}}(d))} }}{V(D1) + V(D2)},$$

- 2) Disease symptom similarity (DSS2)

Human symptom-based disease network (HSDN [49]) was proposed by Zhou et al. The core idea is counting the cooccurrence of disease and symptoms in different literature. In HSDN, each disease can be represented by a vector of symptoms, of which utilizes the inverse document frequency to depict the association strength between symptom and disease. Whereafter, the cosine similarity is adopted to determine the similarity between disease ${d_i}$ and disease ${d_j}$ by leveraging the corresponding vector of symptoms, described below:

4 $$DSS2({d_i},{d_j}) = \cos (ve{c_i},ve{c_j}) = \frac{{\sum\nolimits_x {ve{c_{i,x}} \cdot ve{c_{j,x}}} }}{{\sqrt {\sum\nolimits_x {ve{c_{i,x}}^2} } \cdot \sqrt {\sum\nolimits_x {ve{c_{j,x}}^2} } }}$$

where $ve{c_i}$ represents a vector of symptoms of disease $d_i$.

- 3) Disease Gaussian interaction profile kernel similarity (GIP-D)

Recently, there seems to reach a consensus that GIP kernel similarity performs well in pair-wise association prediction task. Under the inspiration that similar diseases generally show latent patterns with similar microbes [18], we calculated the GIP-D based on the known microbe-disease association matrix A. The equation for this calculation is as below:

5 $$\begin{gathered} GIP{ - }D({d_i},{d_j}) = \exp \left( { - {\eta_d}{{\left\| {{A_c}({d_i}) - {A_c}({d_j})} \right\|}^2}} \right), \hfill \\ {\eta_d} = {{{\eta_d}^{\prime}} \mathord{\left/ {\vphantom {{{\eta_d}^{\prime}} {\left( {\frac{1}{nd}\sum\limits_{i = 1}^{nd} {{{\left\| {{A_c}({d_i})} \right\|}^2}} } \right)}}} \right. \kern-0pt} {\left( {\frac{1}{nd}\sum\limits_{i = 1}^{nd} {{{\left\| {{A_c}({d_i})} \right\|}^2}} } \right)}}, \hfill \\ \end{gathered}$$

Where ${A_c}$ is the *i*th column vector in $A$. Moreover, ${\eta_d}$ is adopted to control the bandwidth and ${\eta_d}^{\prime}$ is usually set as 1 for normalization [50].

#### Microbe similarity network

To collect a broad range of information, we considered multiple perspectives and sources. We not only adopted the GIP similarity, but also utilized the concept of functional similarity, which is recognized in other types of pair-wise known associations. Below are the two types of functional similarity we calculated: DFS1 and DFS2.

- 1) Microbe Gaussian interaction profile kernel similarity (GIP-M)

Similar to GIP-D, the computation difference of GIP-M differs in ${A_c}$, of which was replaced by ${A_r}$ in GIP-M. The subscript r denotes the row in $A$. Moreover, other parameters were kept the same as GIP-D.

- 2) Disease-based functional similarity (DFS1)

Inspired by the calculation method of miRNA functional similarity [48], we computed the DFS1 based on DSS1. To begin with, the similarity score between a disease $d$ and a set of disease $ds$ was calculated as below:

6 $$SS(d,ds) = \mathop {\max }\limits_{{d^i} \in ds} \left( {DSS1(d,{d^i})} \right),$$

The functional similarity value between microbe ${m_x}$ and microbe ${m_y}$ can be derived from the corresponding disease set and the specific equation is described as below:

7 $$DFS1\left( {{m_x},{m_y}} \right) = \frac{{\sum\limits_{d \in d{s_y}} {SS\left( {d,d{s_x}} \right)} + \sum\limits_{d \in d{s_x}} {SS\left( {d,d{s_y}} \right)} }}{{\left| {d{s_x}} \right| + \left| {d{s_y}} \right|}},$$

where $d{s_x}$ and $d{s_y}$ represent the disease sets related to microbe ${m_x}$ and microbe ${m_y}$ in $A$, respectively. Moreover, the operator $\left| {ds} \right|$ denotes the number of elements in the set $ds$.

- 3) Drug-based functional similarity (DFS2)

To calculate DFS2, we focused on the relationship between microbes and drugs and made use of existing databases (MDAD [51] and aBiofilm [52]) for the microbe-drug association prediction task. In the work of predecessors [53], the similarity matrix of drugs had been well calculated. We screened out common microbes between microbe-disease databases and microbe-drug databases and calculated two similarities using the same method as DFS1 from MDAD and aBiofilm. Subsequently, the final DFS2 was computed by averaging the two similarities if the corresponding value of one item is not zero in two databases, and choosing a nonzero item otherwise.

#### Similarity network confusion

In previous works [25, 54], SNF is a commonly used non-linear method that combines multiple similarities to create a unified similarity network. SNF adopted a new normalization method, of which takes self-similarity into consideration. In addition, SNF also computed local affinity for a certain similarity network by the means of K nearest neighbors (KNN). The key step of SNF is iteratively updating the corresponding similarity matrix for each network based on the new normalized matrix and local affinity matrix. Considering that the ability to procure complementary and shared information from multiple sources and robustness to noise, we ultimately utilized SNF to integrate similarities for microbes and diseases, respectively.

##### MVGAEW

**Fig. 5:**
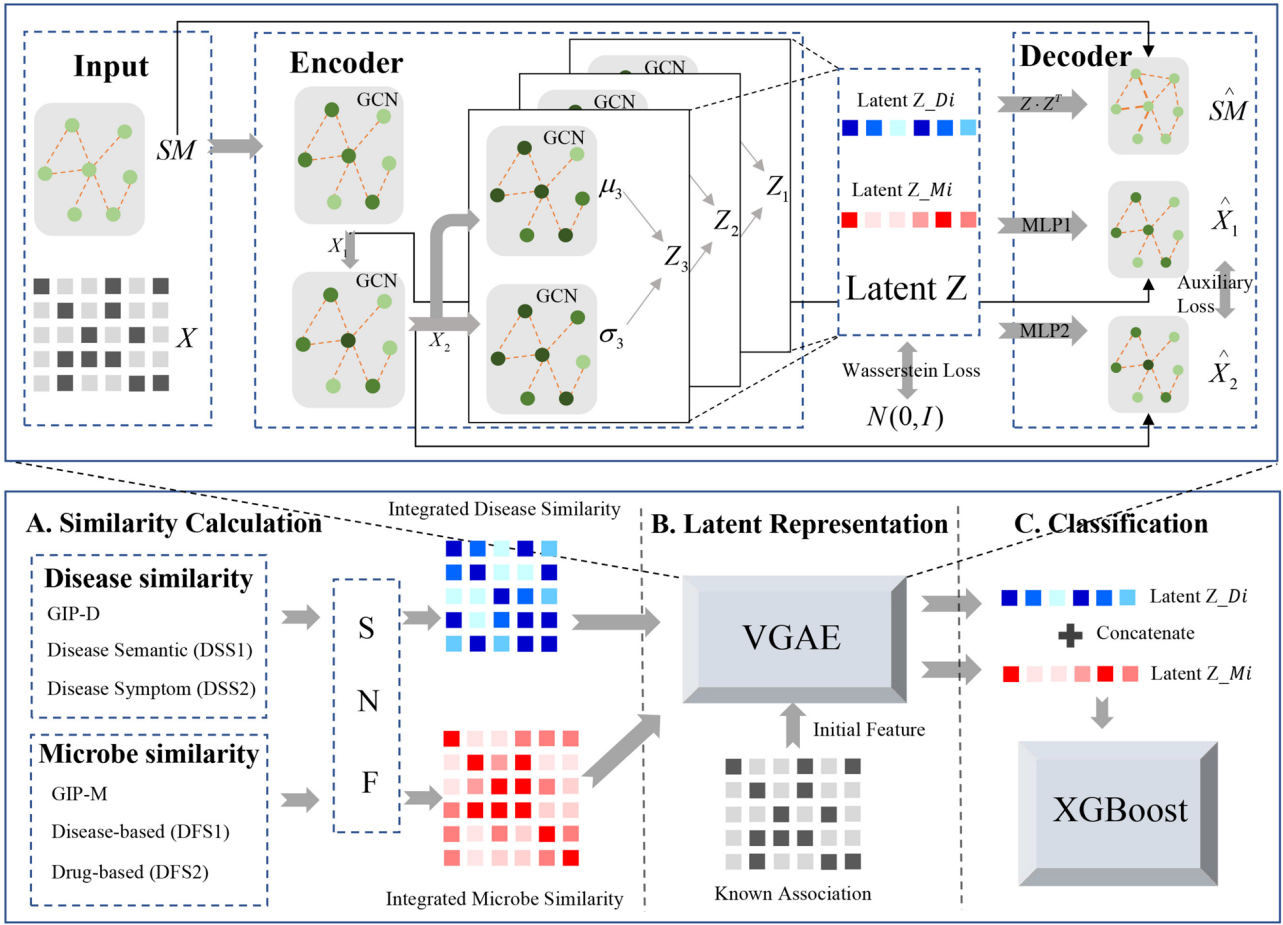
Overall framework of MVGAEW. **A** Calculate and integrate the similarities for microbes and diseases. GIP-D represents the Gaussian interaction profile kernel similarity for disease. DSS1 denotes disease semantic similarity while DSS2 denotes disease symptom similarity. GIP-M is similar to GIP-D, DFS1, and DF2 are functional similarities based on disease and drug, respectively. **B** Adopt an improved VGAE for latent representation with auxiliary tasks. **C** Utilize XGBoost for potential disease-related microbe prediction by inputting the concatenation of latent representation of each microbe and disease

The overall framework of MVGAEW is shown in Fig. 5. We started by integrating similarity matrixes for microbes and diseases using the SNF method. Next, we utilized improved VGAE to represent node embedding based on microbe and disease similarity matrix, respectively. Ultimately, XGBoost was adopted to predict potential disease-related microbes after the concatenation of the latent representation of each microbe and disease. In the stage of latent representation, we designed a multi-scale encoder and decoder with auxiliary tasks to enhance the representational capacity. In addition, we utilized Wasserstein distance to precisely measure two distributions. The main sections of MVGAEW were described as follows:

#### Multi-scale encoder

For convenience, the adjacency matrix was set to the integrated similarity matrix $SM$, while the node features were initialized with the known association matrix $X$. Our encoder including two shared base layers implemented by GCN and a multi-scale variational inference layer, in which two GCNs are supposed to compute the mean $\mu$ and the variance $\sigma$ and then incorporated them as the latent variable $Z$. The output of the first base GCN layer can be represented as:

8 $$\begin{gathered} \overline {X_1} = GCN\left( {X,SM} \right) = ReLU\left( {\overline {S{M_{norm}}} \cdot X \cdot {W_0}} \right), \hfill \\ {\text{where }}\overline {S{M_{norm}}} = {\widetilde D^{ - \frac{1}{2}}} \cdot \overline {SM} \cdot {\widetilde D^{ - \frac{1}{2}}}, \hfill \\ \end{gathered}$$

where $\overline {SM}$ denotes the matrix $SM$ with self-loop, while $\overline{S{M_{norm}}}$ denotes the matrix $\overline {SM}$ processed by symmetrically normalized laplacian matrix. In addition, ${W_0}$ presents the parameters of the GCN model that needs to be learned and $ReLU()$ is a non-linear activation function. Similarly, the output of the second base GCN layer can be represented as:

9 $$\overline {X_2} = GCN\left( {\overline {X_1} ,SM} \right) = ReLU\left( {\overline {S{M_{norm}}} \cdot \overline {X_1} \cdot {W_1}} \right),$$

where ${W_1}$ represents the parameters of the second GCN that needs to be learned. The third multi-scale GCN layer depicts the data distribution by the mean $\mu$ and the log variance $\log \sigma$ as follows:

10 $$\begin{gathered} {\mu_i} = GC{N_\mu }\left( {\overline {X_2} ,SM} \right) = \overline {S{M_{norm}}} \cdot \overline {X_2} \cdot W_\mu^i, \, i \in \left\{ {1,2,3} \right\}, \hfill \\ \log {\sigma_i} = GC{N_\sigma }\left( {\overline {X_2} ,SM} \right) = \overline {S{M_{norm}}} \cdot \overline {X_2} \cdot W_\sigma^i, \, i \in \left\{ {1,2,3} \right\}, \hfill \\ \end{gathered}$$

For *i*th scale layer, the dimension of ${\mu_i}$ and $\log {\sigma_i}$ are consistent, while the dimension between layers differs a lot. Considering calculating the gradient during the backpropagation, we utilized the reparameterization technique to determine the latent variables ${Z_i}$ at different scales, as shown below:

11 $${Z_i} = {\mu_i} + {\sigma_i} * \varepsilon ,$$

where $\varepsilon$ obeys the standard normal distribution $N(0,1)$. By means of concatenation, we obtained the output latent $Z$ as follows:

12 $$Z = {Z_1}|{Z_2}|{Z_3},$$

#### Decoder with auxiliary task

Inspired by the diffusion model [29] and parallel neighborhood reconstruction [27], we innovatively proposed an auxiliary task, multi-order node embedding reconstruction, to enhance the robustness of VGAE. The main decoder is implemented through the inner product between latent variables $Z$ with a sigmoid function to scale the output, as below:

13 $$\widehat {SM} = sigmoid(Z \cdot {Z^T}),$$

To maintain dimensional consistency, we utilized two MLPs to project the dimension of $Z$ into dimensions of ${X_1}$ and ${X_2}$, respectively. The specifics of this process are described below:

14 $$\widehat {X_1} = sigmoid(ML{P_1}(Z)), \, \widehat {X_2} = sigmoid(ML{P_2}(Z)),$$

#### Wasserstein distance

In order to address a common issue where the gradient from KL divergence becomes ineffective or even vanishes during later stages of training [55, 56], we instead employed Wasserstein distance (WD [27, 57]) to substitute KL divergence as the gradient from WD always existed. Accurately measuring the distance between two distributions is critical. While the KL divergence is unsymmetrical, the WD is symmetrical, making it a more suitable choice in some scenarios. In addition, the fabulous property of WD is measuring the distance of two distributions quite well when the degree of overlapping between two distributions is quite low. On the contrary, KL divergence will compute an infinite value. The only shortcoming of WD lies in the demand of large computation, which is often solved by mean of approximation in polynomial time.

For convenience, we used $U$ and $V$ to denote two probability distributions with finite secondary moment defined on $\aleph \in {{\mathbb{R}}^m}$. The optimal mass transportation problem with ${\ell_2}$ transport cost can be solved through 2-Wasserstein distance between $U$ and $V$ defined on $\aleph$ and $\aleph ^{\prime} \in {{\mathbb{R}}^m}$, respectively [58]:

15 $${W_2}\left( {U,V} \right) = {\left( {{{\inf }_{\gamma \in \Gamma (U,V)}}\int_{\aleph \times \aleph ^{\prime}} {\left\| {\aleph - \aleph ^{\prime}} \right\|_2^2d\gamma (\aleph \times \aleph ^{\prime})} } \right)^{{1 \mathord{\left/ {\vphantom {1 2}} \right. \kern-0pt} 2}}},$$

where $\Gamma \left( {U,V} \right)$ denotes the joint distributions of marginals $U$ and $V$. The problem mentioned above can be perceived as a matching problem, and the Hungarian algorithm [59] is well-suited for solving it with the time complexity of $O({n^3})$. In this work, we utilized an efficient algorithm Sinkhorn for approximation, of which adopted a surrogate loss based on continuous relaxation with $O({n^2})$ complexity [60].

#### Loss function

The loss function is formulated below [27, 28]:

16 $$\begin{gathered} L = - {E_{q(Z|SM,X)}}[\log p(\widehat {SM}|Z)] \hfill \\ \, + \frac{1}{M}\sum\limits_{m = 1}^M {\left( {{W_2}[q({Z_m}|SM,X)|p({Z_m})]} \right)} \hfill \\ \, - \frac{1}{2}\sum\limits_{l = 1,2} {{E_{\nu (\overline {X_l} )}}[\log \xi (\widehat {X_l}|Z)]} , \hfill \\ \end{gathered}$$

where $- {E_{q(Z|SM,X)}}[\log p(\widehat {SM}|Z)]$ denotes the binary cross entropy between input similarity network $SM$ and reconstruction similarity network $\widehat {SM}$. The second part represents the loss of WD between all-scale latent representation $q({Z_m}|SM,X)$ and the prior distribution $p({Z_m})\ N(0,I)$. The third part denotes the binary cross entropy between $l$-order node embedding $\overline {X_l}$ and auxiliary node embedding reconstruction $\widehat {X_l}$. In addition, we employed Adam optimizer [61] to minimize the loss function.

#### XGBoost classifier

In this work, we trained an XGBoost model by inputting the concatenation of the latent representations to predict the likelihood between pairs of microbes and diseases. XGBoost [30] is used for supervised learning problems as the classical boosting model in ensemble learning, which is famous for excellent scalability and high efficiency. XGBoost adopted greedy learning through a forward distribution algorithm. In detail, it will learn a CART tree for each iteration to approximate the residuals, which is implemented by a negative gradient between true values and predicted values from the combination model of the previous iteration during training, exactly as other GBDT models. The key point is that XGBoost conducted plenty of optimizations: (1) utilizing the second-order Taylor formula expansion for the optimization of the loss function, which improves its computational accuracy, (2) integrating a regularization term to reduce the form of the objective function and prevent overfitting, (3) adopting blocks storage structure to enables the processing of data in parallel by breaking it down into smaller blocks that can be processed simultaneously on multiple computing units.

## Data Availability

The code of the model and datasets can be downloaded from GitHub (https://github.com/LiangYu-Xidian/MVGAEW, and Zenodo ). All data generated or analyzed during this study are included in this published article, its supplementary information files, and publicly available repositories. For previously published datasets: Ma W, Zhang L, Zeng P, Huang C, Li J, Geng B, Yang J, Kong W, Zhou X, Cui Q. An analysis of human microbe–disease associations. https://academic.oup.com/bib/-article/18/1/85/2562737?login=false#supplementary-data. (2016); Janssens Y, Nielandt J, Bronselaer A, Debunne N, Verbeke F, Wynendaele E, Van Immerseel F, Vandewynckel Y-P, De Tré G, De Spiegeleer B. Disbiome database: linking the microbiome to disease. https://bmcmicrobiol.biomedcentral.com/\-articles/10.1186/s12866-018–1197-5#Sec10 . (2018); Yao G, Zhang W, Yang M, Yang H, Wang J, Zhang H, Wei L, Xie Z, Li W. Microphenodb associates metagenomic data with pathogenic microbes, microbial core genes, and human disease phenotypes. http://www.liwzlab.cn/microphenodb/-#/download. (2020); Skoufos G, Kardaras FS, Alexiou A, Kavakiotis I, Lambropoulou A, Kotsira V, Tastsoglou S, Hatzigeorgiou AG. Peryton: a manual collection of experimentally supported microbe-disease associations. https://dianalab.e-ce.uth.gr/peryton/-#/associations. (2021).

## Abbreviations

SNF: Similarity network confusion
VGAE: Variational graph autoencoder
HMDAD: Human Microbe–Disease Association Database
DSS1: Disease semantic similarity
MeSH: Medical Subject Headings
DAG: Directed acyclic graph
DSS2: Disease symptom similarity
HSDN: Human symptom-based disease network
GIP-D: Disease Gaussian interaction profile kernel similarity
GIP-M: Microbe Gaussian interaction profile kernel similarity
DFS1: Disease-based functional similarity
DFS2: Drug-based functional similarity
KNN: K-nearest neighbors
WD: Wasserstein distance
KL divergence: Kullback–Leibler divergence
CART: Classification and Regression Tree
GBDT: Gradient Boosting Decision Tree
PMID: PubMed IDs
AD: Alzheimer’s disease
CD: Crohn's disease
IBD: Inflammatory bowel disease
CN: Colorectal neoplasms
